# Trends in Adherence to the Mediterranean Diet in Spanish Children and Adolescents across Two Decades

**DOI:** 10.3390/nu15102348

**Published:** 2023-05-17

**Authors:** Estefanía Herrera-Ramos, Laura Tomaino, Almudena Sánchez-Villegas, Lourdes Ribas-Barba, Santiago F. Gómez, Julia Wärnberg, Maddi Osés, Marcela González-Gross, Narcis Gusi, Susana Aznar, Elena Marín-Cascales, Miguel Ángel González-Valeiro, Nicolás Terrados, Josep A. Tur, Marta Segú, Montserrat Fitó, Clara Homs, Juan Carlos Benavente-Marín, Idoia Labayen, Augusto G. Zapico, Jesús Sánchez-Gómez, Fabio Jiménez-Zazo, Pedro Emilio Alcaraz-Ramón, Marta Sevilla-Sanchez, Susana Pulgar-Muñoz, Cristina Bouzas, Clara Sistac-Sorigué, Helmut Schröder, Lluis Serra-Majem

**Affiliations:** 1 Research Institute of Biomedical and Health Sciences (IUIBS), University of Las Palmas de Gran Canaria, 35016 Las Palmas de Gran Canaria, Spain; 2 Centro de Investigación Biomédica en Red Fisiopatología de la Obesidad y la Nutrición (CIBEROBN), Institute of Health Carlos III, 28029 Madrid, Spain; 3 IS-FOOD—Institute for Sustainability & Food Chain Innovation, Universidad Pública de Navarra (UPNA), IDISNA, 31006 Pamplona, Spain; 4 Fundación para la Investigación Nutricional (FIN), Barcelona Science Park, 08028 Barcelona, Spain; 5 Gasol Foundation, Sant Boi de Llobregat, 08830 Barcelona, Spain; 6 CIBER Epidemiology and Public Health (CIBERESP), Carlos III Health Institute, 28029 Madrid, Spain; 7 Hospital del Mar Medical Research Institute (IMIM), 08003 Barcelona, Spain; 8 GREpS, Health Education Research Group, Nursing and Physiotherapy Department, University of Lleida, 25008 Lleida, Spain; 9 EpiPHAAN Research Group, School of Health Sciences, Instituto de Investigación Biomédica en Málaga (IBIMA), University of Málaga, 29590 Málaga, Spain; 10 ImFINE Research Group, Department of Health and Human Performance, Universidad Politecnica de Madrid, 28003 Madrid, Spain; 11 Physical Activity and Quality of Life Research Group (AFYCAV), Faculty of Sport Sciences, University of Extremadura, 10003 Cáceres, Spain; 12 PAFS Research Group, Faculty of Sports Sciences, University of Castilla-La Mancha-Toledo Campus, 45071 Toledo, Spain; 13 Biomedical Research Networking Center on Frailty and Healthy Aging (CIBERFES), 28029 Madrid, Spain; 14 Research Center for High Performance Sport, Catholic University of Murcia, 30107 Murcia, Spain; 15 Facultad de Deporte, UCAM Universidad Católica de Murcia, 30107 Murcia, Spain; 16 Faculty of Sports Sciences and Physical Education, Universida de da Coruña, 15001 A Coruña, Spain; 17 Regional Unit of Sports Medicine-Municipal Sports Foundation of Avilés, 33402 Avilés, Spain; 18 Health Research Institute of the Principality of Asturias (ISPA), 33011 Oviedo, Spain; 19 Research Group of Community Nutrition & Oxidative Stress, IUNICS, University of the Balearic Islands, 07122 Palma de Mallorca, Spain; 20 Health Research Institute of the Balearic Islands (IDISBA), 07122 Palma de Mallorca, Spain; 21 Probitas Foundation, 08022 Barcelona, Spain; 22 Global Research on Wellbeing (GroW), Faculty of Health Sciences, Blanquerna Ramon Llull University, 08025 Barcelona, Spain; 23 Department of Language, Arts and Physical Education, Universidad Computense de Madrid, 28040 Madrid, Spain; 24 Preventive Medicine Service, Canarian Health Service, Centro Hospitalario Universitario Insular Materno Infantil (CHUIMI), 35016 Las Palmas de Gran Canaria, Spain

**Keywords:** Mediterranean diet, children, adolescents, nutrition survey, Spain

## Abstract

Unhealthy dietary habits determined during childhood may represent a risk factor to many of the chronic non-communicable diseases (NCDs) in adulthood. Mediterranean Diet (MD) adherence in children and adolescents (8–16 years) living in Spain was investigated using the KIDMED questionnaire in a comparative analysis of two cross-sectional nationwide representative studies: enKid (1998–2000, *n* = 1001) and PASOS (2019–2020, *n* = 3540). Taking into account the educational level of pupils, as well as the characteristics of the place of living, a significant association was found between a KIDMED score ≥ 8 (optimal MD adherence) and primary education as well as residency in an area of <50,000 inhabitants, while living in the southern regions was associated with non-optimal MD adherence (*p* < 0.001). Participants of the 2019–2020 study showed an increase in the consumption of dairy products (31.1% increase), pasta/rice (15.4% increase), olive oil (16.9% increase), and nuts (9.7% increase), as well as a decreased sweets and candies intake (12.6% reduction). In contrast, a significantly lower MD adherence was found when comparing the 2019–2020 (mean ± SE: 6.9 ± 0.04) and the 1998–2000 study (7.37 ± 0.08); *p* < 0.001), due to less consumption of fish (20.3% reduction), pulse (19.4% reduction), and fruits (14.9% reduction), and an increased intake of commercial goods/pastries or fast-food intake (both 19.4% increase). The lowest adherence was recorded for adolescents also in the most recent study, where 10.9% of them presented a KIDMED score ≤ 3. This study shows that eating habits are deteriorating among Spanish children and adolescents. Such findings point out the urgency of undertaking strong measures to promote the consumption of healthy, sustainable, and non-ultra-processed food, such as those available in an MD, not only at a scientific and academic level, but also at a governmental one.

## 1. Introduction

The Mediterranean diet (MD) is one of the world’s leading healthy dietary models. It includes individual foods that are particularly beneficial to health, such as vegetables, fruits, legumes, cereals, fish, nuts, extra virgin olive oil. The diet is also limited in sugars and free of trans fats, and even the way they are prepared, combined, and/or consumed has beneficial effects on environmental sustainability [1,2,3]. Improving dietary habits to align with the MD seems to be associated with higher physical fitness and PA in youth, lower sedentary behaviours, and better health in general [4].

Robust evidence shows that high adherence to the Mediterranean dietary pattern is associated with a reduced risk of cardiovascular events [5], type 2 diabetes [6], cancer [7], and other non-communicable diseases. Moreover, higher adherence to the Mediterranean diet during pregnancy is related to a lower risk of preterm birth and to better offspring cardiometabolic health [8]. An inverse linear dose–response relation was also observed between MD adherence and the risk of all-cause mortality [9]; according to a systematic analysis for the Global Burden of Disease Study 2017, dietary factors (i.e., high intake of sodium, low intake of whole grains, and high sugar-sweetened beverage consumption) were related to the death of about 11 million people globally between 1997 and 2017, and if healthy growth of children and adolescents is to be addressed as a priority, it is mandatory to analyze their dietary patterns [10].

The KIDMED questionnaire is a dichotomous response options (Yes/No) questionnaire created to estimate adherence to the Mediterranean diet in children and young adults, based on the principles that sustain Mediterranean dietary patterns and those that undermine it [11]. It has been extensively used to assess the children and adolescents’ MD adherence [12] in different areas of the world [13], especially Europe [14] and Spain [15,16,17,18,19,20]. enKid was essential to validate it [11], a cross-sectional study designed to determine the nutritional status, eating habits, lifestyle, and family conditions of a representative sample of the Spanish population aged 2–25 years. The findings of this study (1998–2000) showed the risks of deterioration of the MD adherence in the Spanish youth population just before the beginning of the 21st century. Despite the worrying findings observed, to date, no other study comparing two large and representative surveys regarding adherence to MD changes in 8–16 individuals have been published. It would be useful to know how the diet quality has changed over time in children and adolescents living in Spain. At the end of the second decade of this new century (2019–2020), the PASOS (Physical Activity, Sedentarism, lifestyles and Obesity in Spanish youth) study has been carried out. It was a cross-sectional study representative of the Spanish population aged 8–16 years, in which the KIDMED questionnaire was used to evaluate adherence to the MD [21].

The aim of the present study is to analyze the differences in terms of MD adherence over the last 20 years in children and adolescents living in Spain by comparing the enKid (1998–2000) and PASOS (2019–2020) samples, and to identify the factors or conditions that could influence these changes.

## 2. Materials and Methods

### 2.1. Study Design, Setting and Participants

This study involved a comparison of two national and representative studies, which are described below.

#### 2.1.1. 1998–2000 Study

enKid was a multicenter, cross-sectional, population-based study conducted by face-to-face interview, and it aimed to evaluate dietary habits and nutritional status of Spanish schoolchildren and adolescents. Baseline data were collected from May 1998 to April 2000. The study sample was derived from residents registered in the Spanish official population census, aged 2–24 from the different regions (in Spain called “autonomous communities”) of Madrid, Castilla y León, Castilla-La Mancha, Extremadura, Catalonia, Aragon, the Balearic Islands, Galicia, Asturias, Cantabria, the Basque Country, Navarra, La Rioja, Andalucia, Valencia, Murcia, and the Canary Islands, for a total sample size of *n* = 3534. Home interviews were carried out by dietitians or nutritionists who attended and completed a training process specific for this study purpose [22]. For children aged 6–13 years, the interviews were answered by the children themselves, with the support of the caregiver responsible for his/her dietary intake. Survey data were entered in laptop computers by the staff who carried out the interviews, with a specifically designed software. Completed interview data were periodically sent to the coordinating centers in Barcelona and Bilbao. To recover missing data, additional information was obtained from school lunch menus or telephone interviews with the food service director of the selected school. For our study purpose, enKid study participants aged 8–16 years old were selected and those without answer to all KIDMED questions were excluded since answering all questions is a condition to obtain KIDMED scores. Finally, the sample size was *n* = 1001 with 505 males (50.4%) and 617 individuals (61.6%) attending secondary school.

#### 2.1.2. 2019–2020 Study

The PASOS (Physical Activity, Sedentarism and Obesity in Spanish Youth) study is a multicenter, cross-sectional, population-based study (trial registration number: ISRCTN34251612), with a protocol that has been published and is available elsewhere [21]. The cohort study, aimed to determine physical activity levels and the lifestyle factors associated with it in Spanish children and adolescents, was coordinated by the Gasol Foundation. Field and scientific work were performed together with 13 highly experienced research groups working at universities and research centers in several regions of Spain. It was designed for a total of 3994 participants (1997 participants in each of the two following age groups: primary school (8–11 years old) and secondary school (12–16 years). This categorization was performed to achieve a statistical power of ≥80% to identify a population increase of 8% as significant (*p* ≤ 0.05). The number of municipalities was 121 and the final sample size was 4508 participants. Baseline data were collected from March 2019 to February 2020, in a total of 242 classrooms of primary and secondary public schools. Two visits were carried out in each school by two study investigators with a background in physical education, nutrition or other health sciences, who completed a 1-day training session on the project methodology, hosted by the Gasol Foundation. Anonymized participants’ variables were collected during school hours, using an online system for the questionnaires (Qualtrics©) regarding dietary habits, sleeping time, parental educational level, and the logistical help of teachers to organize sessions for variables’ collection. Written informed consent was obtained from all children’s parents or caregivers after inviting them to participate in the study. Completed survey data were sent electronically from all participating research groups to the coordinating center in Barcelona. PASOS study participants aged between 7.9 and 17.1 years who answered all KIDMED questions were selected for this study. Finally, the sample size was *n* = 3540, with 1707 males (48.2%) and 1724 individuals (48.7%) attending secondary school.

### 2.2. Dependent Variable

#### Mediterranean Diet Pattern Adequacy

The MD quality was measured using the KIDMED index, a validated tool utilized on a total of 3850 children and youths aged 2–24 years and representative of the Spanish population, for evaluating the degree of adherence to the Mediterranean dietary pattern [11]. The KIDMED index consists of 16 questions with dichotomous answer: items with a “yes” answer positively related with the MD scored +1 (i.e., 12 items), and items with “yes” answers negatively related to the MD scored −1 (i.e., 4 items). Items with a “no” answer scored 0. As a result, the total score ranges from −4 to 12. In the present study, adherence to the MD of both studies (1998–2000 and 2019–2020) was classified into three levels based on KIDMED scores: 8–12 (optimal/good adherence), 4–7 (average/medium adherence), and ≤3 (very poor adherence/poor). In addition, it was classified as a dichotomous variable: 8–12 (optimal/good adherence) and ≤7 (medium/poor adherence).

### 2.3. Independent Variables

#### 2.3.1. Residential Place Characteristics

Participants were classified according to their region of residence in Spain as follows: north, center or south. Northern regions included the following autonomous communities: Galicia, Principado de Asturias, País Vasco, Comunidad Foral de Navarra, La Rioja, Aragón, Cataluña, and Islas Baleares. Central regions included the following communities: Castilla La Mancha, Castilla y León, Comunidad de Madrid, and Extremadura. Southern regions included the following communities: Andalucía, Canarias, Comunidad Valenciana and Región de Murcia. Moreover, participants were classified into four groups according to the population density of the municipality of their educational center: a mean population of more than 350,000 inhabitants; between 50,000 and 350,000 inhabitants; between 50,000 and 10,000 inhabitants; and less than 10,000 inhabitants. Additionally, a dichotomous variable was computed for the following scenario: more than 50,000 inhabitants and less than 50,000 inhabitants.

#### 2.3.2. Parental Education Level

Each study participant provided two sets of questionnaires filled-in separately by one or both parents/legal guardians. Parents’ sex and educational level were categorized as a dichotomous variable as follows: university (if at least one of the adults responsible for the child had a university education) and non-university (if none of the adults responsible for the child had a university degree).

#### 2.3.3. Sleeping Hours

According to the National Sleep Foundation, children between the ages of 6 and 13 should get between nine and eleven hours of sleep. Adolescents between 14 and 17 should get eight to ten hours of sleep. The Sleep Habits Survey for Adolescent [23] was performed to assess sleep quality and rest time. Sleep recommendation adherence was calculated for each participant. Due to missing data, the final sample size available for this variable was 994 and 3506 based on the 1998–2000 and 2019–2020 studies, respectively.

### 2.4. Statistical Analysis

Participants were stratified into four subgroups by sex, age group, school level (primary and secondary), and demographic characteristics (population density and geographical area of residence). Data are presented as mean ± standard deviation (SD). MD adherence level was stratified by categorical variables (sex, age, residential place characteristics, and parental education level), which were expressed as mean values with CI95%. Continuous variables were tested for normality using the Kolmogorov–Smirnov test. The Student χ2 test was carried out to compare sociodemographic characteristics between studies. The Mann–Whitney U test was used to compare the KIDMED mean scores between the 1998–2000 and 2019–2020 surveys. Multivariate logistic regression was performed to identify the potential determinants of a good scoring in the KIDMED (≥8), according to different variables initially identified by univariate logistic regression. All statistical analysis was performed using SPSS for Windows version 22 (SPSS, Inc., Chicago, IL, USA). Figures were generated with RStudio version 4.2.2.

## 3. Results

### 3.1. Comparison of Sociodemographic Characteristics of the Two Studies

The sex distribution was similar in the two studies, with 49.4% and 51.0% males attending primary school and 50.1% and 47.4% attending secondary school, for the 1998–2000 and 2019–2020 surveys, respectively. In the 1998–2000 study, 47.4% of the primary school children and 56.2% of secondary school children lived in a place with less than 50,000 inhabitants, while in the 1998–2000 study, the percentages were higher (62.7% and 66.4%, respectively). The percentage of participants from the northern areas of Spain in the 1998–2000 survey was high in the two educational grades (51.8% and 46.8%, respectively), while in the 2019–2020 study, the distribution by region was wider, and therefore, the percentage of participants from the north was lower (38.5% and 39%, respectively).

### 3.2. Adherence to the Mediterranean Diet

**Figure 1:**
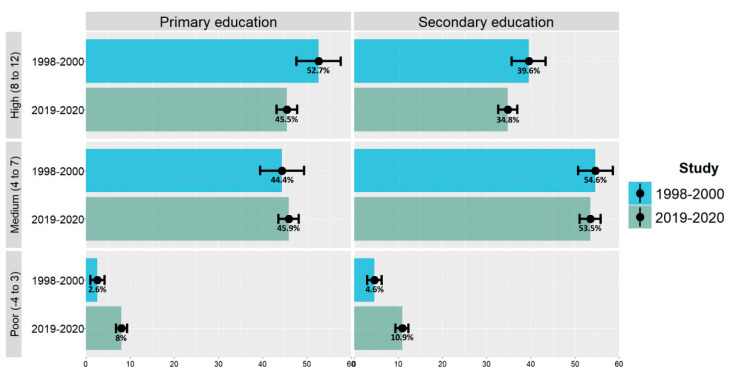
Time trends of KIDMED score categories in Spanish population studies.

Figure 1 shows time trends of the MD adherence, classified into three levels based on KIDMED scores and educational stage of participants in both studies. The KIDMED score was lower in those attending secondary school: 60% of the participants in the 1998–2000 survey and 64.8% in the 2019–2020 survey scored < 8 points. The lowest adherence was recorded for adolescents in the PASOS study, where 10.9% of them presented a KIDMED score ≤ 3.

**Figure 2:**
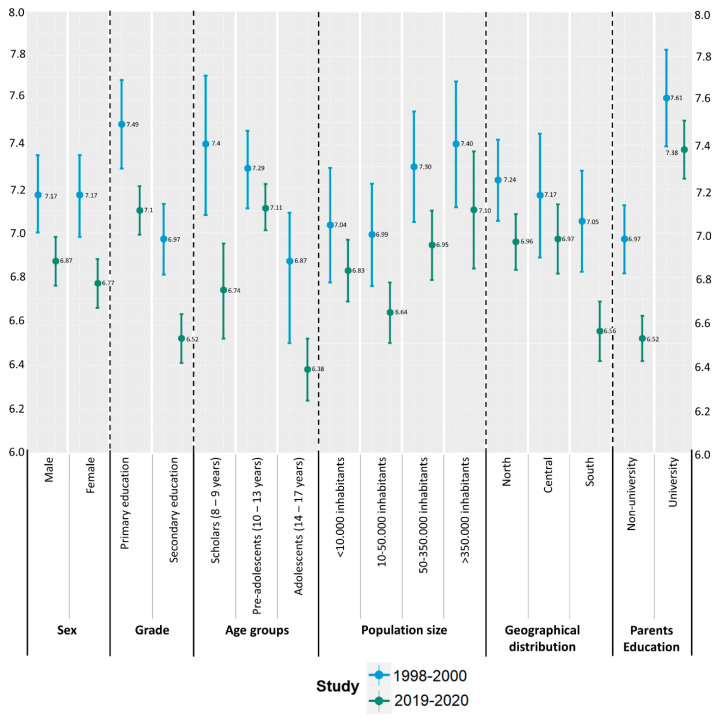
Comparison of the total KIDMED 16-items scores between the 1998–2000 and 2019–2020 surveys.

As shown in Figure 2, in 1998–2000, overall, higher mean scores were obtained in the KIDMED test compared to those in 2019–2020, in all the categories investigated (related to the characteristics of the participants, the geographical area where they lived, and their parents’ education level). Across the two decades, significant differences (*p* < 0.001) in KIDMED mean scores for Spanish children and adolescents were observed for both sexes (male/female) and educational grades (primary/secondary), for younger children (aged 8 to 9 years), for those living in the south region of Spain, and for children and adolescents whose parents did not have a university degree. In the 1998–2000 cohort, the KIDMED test result was (mean ± SE) = 7.37 ± 0.08, while in the 2019–2020 sample, it was 6.96 ± 0.04.

Table 1 shows the association between the adherence to the MD, classified in three groups according to the KIDMED score (poor if ≤3, medium if 4–7, and high if ≥8) and selected variables, i.e., sex, school grade, age group, population density, geographical characteristics of the place of living, and parental educational level. Overall, 219 participants attending secondary school (particularly, those aged 14 to 17 years) had poorer adherence to a MedDiet, as shown by the KIDMED tests, than younger schoolers. Both, in the 1998–2000 and 2019–2020 samples, better adherences to the MD scores were associated with those participants living in the northern regions of Spain (*p* < 0.001).

A multivariate logistic regression selecting a KIDMED score ≥ 8 as the dependent variable was carried out to identify the potential factors associated with the adherence to the MD in 2019–2020. Among study participants, significant differences in terms of MD adherence were observed, adjusting for the following selected variables: sex, schooler’s grade, parents’ education, and country regions. The results show a significant association between a KIDMED score ≥ 8 and primary education (OR 1.680, 95% CI, 1.35–2.09). Similarly, having the residency in an area of <50,000 inhabitants was positively associated with higher levels of adherence to the MD (OR 1.83 95%CI, 1.47–2.28), while living in the southern regions was associated with lower KIDMED scores (OR 0.66, 95% CI, 0.51–0.85).

Table 2 shows that in 1998–2000 a minimum KIDMED score equal to 1 was found, while in 2019–2020, the minimum KIDMED score was −3 (i.e., very poor diet). Adolescents, females, and those living in the southern regions of Spain showed lower adherence to MD two decades later after Bonferroni correction. Finally, analysing the parents/legal guardians’ education, we observed that diet quality worsened in those children and adolescents whose parents’ education was non-university, as reported in Table 2.

### 3.3. Food Habits

As shown in Table 3, compared to the years 1998–2000, in the 2019–2020 study, the following results were observed: a 19.4% increase in the consumption of two types of highly processed foods (i.e., refined foods or pastries and fast-food), a 20.3% reduction in the consumption of fish, a 19.4% reduction in legumes and a 14.9% reduction in daily fruit. Nevertheless, a 31.1% increase of dairy products consumption occurred along the day (although an 8.1% reduction in dairy products consumption was observed for breakfast.) Additionally, a 16.9% increase in the use of olive oil at home, a 15.4% increase of rice or pasta consumption, and a 7.2% increase in the regular consumption of fresh or cooked vegetables was observed. Across the two decades, the adherence to MD was low, and it was observed that adolescents presented the lowest adherence in both surveys. An overall worsening of the diet quality was registered, especially in terms of higher consumption of refined foods, pastries, and fast-foods, acting together with a lower intake of fish, legumes, and fruit. In particular, the findings of this study show an overall reduction in daily fruit consumption: 91.3% of males attending primary school in 1998–2000 consumed fruit or fruit juice every day, which dropped to 75.4% in 2019–2020. Similar figures for males at secondary school were observed (from 85.4% to 68.3%). Females, as well, presented a reduction in daily fruit consumption, from 89.9% of those attending primary school in 1998–2000 to a 77.1% in 2019–2020, and from 85.7% attending secondary school to 69.3% two decades later. Almost a half of secondary schoolers had a second fruit every day in 1998–2000, but a reduction was observed in 2019–2020, where only 41.7% of males and 43.1% of females consumed a second piece of fruit. In addition, fish consumption presented a reduction from 1998–2000 to 2019–2020.

In 1998–2000, among children attending primary school, 78.6% of males consumed fish at least 2–3 times per week, while after two decades, the percentage dropped to 65.4%. Similar findings were observed for females (from 85.1% to 66.1%). These figures were observed also for secondary schoolers, as shown in Table 4. In the 1998–2000 cohort, more than 90% of primary schoolers consumed pulses more than once a week, while in the 2019–2020 cohort, the percentages reduced to 68.7% in males and 69.6% in females. Similar findings were observed for children and adolescents attending secondary school.

The consumption of pasta and rice, on a daily basis, was higher in 2019–2020 than 1998–2000, but without achieving the 50% of participants, except for males attending secondary school in the 2019–2020 cohort. An alarming result, from 1998–2000 to 2019–2020, was the higher consumption of refined baking products for breakfast and fast-food meals observed at both educational levels, and in females and males. In the 2019–2020 study, almost 1/3 of participants overall had commercially baked goods or pastries for breakfast. In 1998–2000, among primary schoolers, less than 1.6% of females reported to consume fast-food one or more times a week, but these figures increased more than 20% in 2019–2020. Moreover, in the 2019–2020 sample, 25.1% of male adolescents consumed fast-food once a week, while this percentage amounted to just 4.9% in 1998–2000.

**Figure 3:**
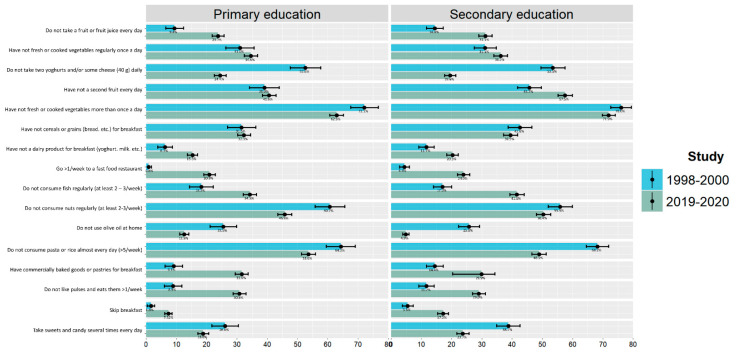
Comparison of negative answers to the KIDMED test among the 1998–2000 and 2019–2020 surveys by scholar grade: primary (P) and secondary (S) education.

Figure 3 shows the answers to the KIDMED test items scoring 0 (items in favour of a high MD) or −1 (items against a high MD) in the 2019–2020 study and 1998–2000 survey of participants attending primary and secondary school, respectively. A difference of diet quality in terms of daily fruit intake as well as fish and pulses consumption is evident in both groups across the two decades, observed together with a higher consumption of commercially baked goods, pastries, and fast-food, with this difference being more prominent in those participants attending primary school. Moreover, the percentage of children and adolescents who do not have breakfast is higher in 2019–2020 for both educational grades than in 1998–2000. Conversely, a higher olive oil consumption at home was observed in 2019–2020 compared to 1998–2000 in all study participants, as well as a lower consumption of candies and sweets. Furthermore, the consumption of cheese and/or dairy products was higher in 2019–2020 than in 1998–2000.

### 3.4. Sleep Hours

As shown in Table 1, in the 1998–2000 study, the majority of participants complied with the adequate rest recommendations, while 12% of the study participants did not comply with the recommendations of sleeping hours per day. However, in the 2019–2020 study, these percentages were higher, as 28.3% of the participants presented a lower number of sleeping hours per day than the minimum limit indicated by the National Sleep Foundation. As shown in Table 1, in the 2019–2020 study, the number of participants that complied with sleep recommendations independently of the degree of adherence to the MD was lower than in 1998–2000. It is worth mentioning that 45.9% of participants who did not comply with adequate sleep recommendations have poor adherence to the MD.

## 4. Discussion

The findings of this study show a significant worsening of dietary habits among Spanish children and adolescents, as measured by the KIDMED index using a comparison of the enKid (1998–2000) and PASOS (2019–2020) surveys, mainly represented by higher consumption of refined foods, pastries, and fast-foods, observed together with a lower intake of fish, legumes, and fruit, in 2019–2020 compared to 1998–2000. These findings represent a worrying indicator regarding the diet quality of a representative cohort of the current young Spanish population.

The adherence to MD is influenced by multiple factors, including non-modifiable ones, such as socioeconomic status and food price [24] and modifiable elements, such as behavioural patterns [25,26]. The dietary pattern to which children and adolescents, as well as adults, adhere is the result of the interaction between the above-mentioned factors, as well as personal preferences, culture, values, their physical and social environment, but also the economic situation and their commercial environment. The findings of this study show that lifestyle aspects, such as adequate sleeping time, were positively associated with a better MD adherence, which are conclusions reached with similar studies on the issue [27,28]. Moreover, it has confirmed that children’s and adolescents’ level of adherence to the MD has decreased in Spain over the last two decades.

The food consumed by children and adolescents will depend on what is available in their cultural, family, educational, and leisurely environments. Moreover, most of the factors influencing dietary pattern have undergone major changes in recent decades due to constant and rapid transformation in the Western economy, increased urbanization, accentuated increase in technology, as well as globalization of food production and consumption [10]. In the European Union, countries still face relevant challenges to regulate the unhealthy food and beverage advertising addressed to children and adolescents. Most of ads do not coincide with the nutrient profile model of WHO because these ads are focused on foods with high caloric content, low nutritional quality, elevated content of sugars and salt, and a scarce or null content of fruits and vegetables. In addition, the most advertised foods are sugary breakfast cereals, confectionery products, salty snacks with high fat content, soft drinks, and fast-food restaurants [29], a sector that in Spain has twice as many establishments as it had 20 years ago [30]. In our study, the results of the 1998–2000 and 2019–2020 data comparison show an increase of 26 and 5 times in the KIDMED item regarding “fast-food” in both primary and secondary education participants, respectively. Our data confirm that more than 20% of Spanish children and adolescents (2019–2020 study participants) go to fast-food restaurants once or more times per week.

Compared to two decades ago, Spanish children and adolescents present eating habits which are detrimental for health and inadequate to ensure a high-quality diet. The overall suboptimal MD adherence (KIDMED score ≤ 8) in Spaniards aged 8–17 years underlined by the findings of this study, and its worsening trend observed from 1998–2000 to 2019–2020, should be considered an issue of major concern. To date, the mean consumption of fruits and vegetables overall is well below the recommended values in adolescents, and the pattern is shifting towards a diet richer in saturated fats, refined cereals, simple carbohydrates, and processed foods [11]. The results of the present study corroborate this trend; nevertheless, there is an improvement in the use of olive oil at home, an increase in the frequency of consumption of nuts and rice or pasta weekly, an increase in the vegetables consumption, and an increasing in dairy products throughout the day (but not at breakfast).

Healthy habits help the maintenance of good physical, cognitive, psychological, and social development in the early stages of life, and are not exclusively governed by nutrition-related aspects, but other factors also have a role, i.e., the lifestyle itself (sleeping time, physical activity, or the use of screens); the conditions of the family environment (such as the degree of education of the parents) [20]; and the characteristics of the living place (population size, degree of urbanization). Our data shows that a lower parental educational level is associated with poorer adherence to the MD. This can have a double explanation: On the one hand, it could be due to a lower purchasing power of the family itself since the MD is considered more expensive than the Western diet. On the other hand, a lower cultural level and awareness regarding the benefits of a healthy diet might play a role. Moreover, some evidence suggests that those adolescents who consumed their meals at home with their family entourage were less likely to eat unhealthy foods [31]. Of note, overall, and especially in low-income areas, mortality associated with dietary factors is higher [10]. In fact, unhealthy eating represents a risk factor for many of the non-communicable diseases that are prevalent in adult population both in Europe and Western countries [32,33]. In the meantime, during childhood and adolescence, being overweight is considered one of today’s major concerns because of its negative impact on long-term health [34]. The burden of an unhealthy diet during childhood and adolescence will have important consequences in terms of non-communicable diseases and obesity-related conditions but also on governments’ healthcare expenditure. Therefore, the findings of the present study should be taken into careful account, not only by public health and preventive medicine professionals but also by policymakers and stakeholders.

As childhood and adolescence represent a key period during which eating habits and behaviours are developed and established, habits that often continue into early adulthood [34], the MD should be considered an ally [35] and a valid tool for the maintenance of good health status and for the prevention on many NCDs. Proper nutritional education at an early age promotes the establishment of healthy eating patterns [36]. Nutrition education in schools is considered useful in improving knowledge about nutrition, but few studies suggest that it is effective in altering eating behaviours in the absence of environmental and structural changes [37]. There is evidence that diet quality in childhood and adolescence is associated with health-related outcomes later in life; however, the causal relationship between diet quality and NCDs is not well established, as there may be other factors that influence both diet quality and NCD risk, such as socioeconomic status, physical activity, genetics, and environmental exposures [38]. Therefore, a change in diet quality may have different implications for future risk of NCDs, depending on the direction, magnitude, duration, and timing of the change, as well as the individual characteristics and context of the children and adolescents. Improving diet quality by increasing the intake of fruits, vegetables, whole grains, and healthy fats may reduce the risk of NCDs by lowering blood pressure, cholesterol, inflammation, and oxidative stress [38]. On the other hand, worsening diet quality by consuming more processed foods, added sugars, saturated fats, and sodium may increase the risk of NCDs by promoting obesity and chronic inflammation. However, these effects may not be immediate or linear, as NCDs develop over a long period of time and may be influenced by other factors throughout the life course. Therefore, it is important to monitor diet quality and NCD risk factors regularly, and to intervene early to prevent or delay the onset of NCDs in children and adolescents.

## 5. Conclusions

Adherence to the MD has decreased among Spanish children and adolescents in the last 20 years. It is advisable to undertake strong measures to promote the consumption of healthy foods whose consumption has been significantly reduced, especially fish, legumes, and fruits, and to maintain the improvement observed in olive oil and nut intake and the reduction of sweets and candies consumption. The increase in eating at fast-food restaurants more than once a week and commercially baked goods and pastries consumption are considered as additional negative outcomes of this study, and contribute to move children and adolescents away from the MD. This study shows that eating habits are deteriorating among Spanish children and adolescents. Such findings point out the urgency of ensuring a high-quality diet. It is advisable to undertake strong measures to promote the consumption of healthy, sustainable, and non-ultra-processed food, such as those available in a MD, not only at a scientific and academic level but also at a governmental one. It is important that Spanish children and adolescents follow a Mediterranean diet because it has many benefits for their health and quality of life. Some of these benefits are the higher intake of essential nutrients that contribute to proper functioning of the body and the physical and intellectual development; a greater variety and balance in food that favors the enjoyment of food and the development of healthy habits from childhood; and a greater adaptation to the environment and the Mediterranean culture, which implies an active, social, and sustainable lifestyle. Therefore, an important, multidisciplinary effort, including public and private sectors, should be made to reverse this worrying trend towards an unhealthy diet among Spanish children and adolescents.

## Figures and Tables

**Table 1:** Comparison of the degree of adherence to the Mediterranean diet (KIDMED Index score) between the two studies according to the selected variables.

	Adherence to Mediterranean Diet (KIDMED Index Categories)														
	Poor ≤ 3					Medium 4–7					High ≥ 8				
	1998–2000		2019–2020		*p*	1998–2000		2019–2020		*p*	1998–2000		2019–2020		*p*
	*n*	%	*n*	%		*n*	%	*n*	%		*n*	%	*n*	%	
Sex															
Male	18	46.2	154	46		258	50.4	841	47.6		229	50.9	712	49.5	
Female	21	53.8	181	54	-	254	49.6	927	52.4	-	221	49.1	725	50.5	-
School grade															
Primary education	10	25.6	147	43.9		171	33.4	838	47.4		203	45.1	831	57.8	
Secondary education	29	74.4	188	56.1	<0.05	341	66.6	930	52.6	<0.001	247	54.9	606	42.2	<0.001
Age group															
(8–10]	5	13.2	77	23.1		71	14.1	273	15.5		84	18.9	241	16.9	
(10–12]	4	10.5	56	16.8		100	19.8	471	26.8		106	23.8	499	35.1	
(12–14]	14	36.8	68	20.4		159	31.5	402	22.9		139	31.2	339	23.8	
(14–17]	15	39.5	132	39.6	-	174	34.5	613	34.8	<0.001	116	26.1	344	24.2	<0.001
Population size															
<10,000 inhabitants	10	25.6	83	24.8		132	25.8	553	31.3		105	23.3	425	29.6	
10–50,000 inhabitants	11	28.2	136	40.6		159	31.1	630	35.6		112	24.9	462	32.2	
50–350,000 inhabitants	10	25.6	92	27.5		132	25.8	435	24.6		124	27.6	409	28.5	
>350,000 inhabitants	8	20.5	24	7.2	<0.05	89	17.4	150	8.5	<0.001	109	24.2	141	9.8	<0.001
Population size															
<50,000 inhabitants	21	53.8	219	65.4		291	56.8	1183	66.9		217	48.2	887	61.7	
>50,000 inhabitants	18	46.2	116	34.6	-	221	43.2	585	33.1	<0.001	233	51.8	550	38.3	<0.001
Geographical distribution															
North	17	43.6	112	33.4		245	47.9	677	38.3		226	50.2	582	40.5	
Centre	11	28.2	80	23.9		109	21.3	432	24.4		97	21.6	394	27.4	
South	11	28.2	143	42.7	-	158	30.9	659	37.3	0.001	127	28.2	461	32.1	0.001
Geographical distribution															
Centre	11	28.2	80	23.9		109	21.3	432	24.4		97	21.6	394	27.4	
Northeast	11	28.2	69	20.6		91	17.8	419	23.7		128	28.4	342	23.8	
North	6	15.4	43	12.8		154	30.1	258	14.6		98	21.8	240	16.7	
South and Canary Islands	6	15.4	90	26.9		103	20.1	364	20.6		80	17.8	263	18.3	
Levante	5	12.8	53	15.8	-	55	10.7	295	16.7	<0.001	47	10.4	198	13.8	<0.001
Comply with sleep recommendations															
Yes	33	84.6	178	54.1		450	88.2	1206	68.7		392	88.1	1130	79.5	
No	6	15.4	151	45.9	<0.001	60	11.8	550	31.3	<0.001	53	11.9	291	20.5	<0.001
Parental Education															
Non-university	32	82.1	251	79.2		373	72.9	1131	68.1		287	63.8	773	56.5	
University	7	17.9	66	20.8	-	139	27.1	531	31.9	-	163	36.2	594	43.5	<0.01

**Table 2:** Comparison of KIDMED Index scores among the periods 1998–2000 and 2019–2020 according to the selected variables.

	Adherence to Mediterranean Diet (KIDMED Index Scores)								
	1998–2000				2019–2020				
	*n*	Mean ± SD	Min	Max	*n*	Mean ± SD	Min	Max	*p*
Sex									
Male	505	7.17 ± 2.04	1	12	1707	6.87 ± 2.39	−3	12	0.024
Female	496	7.17 ± 2.06	2	12	1833	6.77 ± 2.44	−2	12	0.002
Grade									
Primary education	384	7.49 ± 1.98	2	12	1816	7.10 ± 2.41	−2	12	0.012
Secondary education	617	6.97 ± 2.07	1	12	1724	6.52 ± 2.38	−3	12	0.00005
Age groups									
Scholars (8–9 years)	159	7.40 ± 1.99	2	12	589	6.73 ± 2.63	−1	12	0.008
Pre-adolescents (10–13 years)	520	7.29 ± 2.07	2	12	1835	7.11 ± 2.32	−2	12	-
Adolescents (14–17 years)	322	6.87 ± 2.02	1	11	1116	6.38 ± 2.38	−3	12	0.001
Academic Year									
3° primary degree	68	7.38 ± 2.02	3	11	353	6.73 ± 2.58	−1	12	-
4° primary degree	88	7.35 ± 2.07	2	12	395	6.89 ± 2.54	−1	12	-
5° primary degree	124	7.68 ± 1.83	2	12	544	7.44 ± 2.21	−1	12	-
6° primary degree	104	7.45 ± 2.03	2	11	523	7.16 ± 2.34	−2	12	-
1° secondary degree	137	7.12 ± 2.16	2	11	370	7.03 ± 2.39	1	12	-
2° secondary degree	170	6.96 ± 2.07	2	11	380	6.49 ± 2.26	−1	12	0.025
3° secondary degree	174	6.83 ± 2.05	2	11	450	6.48 ± 2.39	−3	12	-
4° secondary degree	136	7.01 ± 2.03	1	12	525	6.22 ± 2.40	1	12	0.00032
Population size									
<10,000 inhabitants	247	7.04 ± 2.07	2	12	1061	6.83 ± 2.32	−1	12	-
10–50,000 inhabitants	282	6.99 ± 1.98	2	11	1228	6.64 ± 2.45	−1	12	0.045
50–350,000 inhabitants	266	7.30 ± 2.07	1	12	936	6.95 ± 2.45	−3	12	-
>350,000 inhabitants	206	7.40 ± 2.07	2	12	315	7.10 ± 2.39	1	12	-
Spain region									
North	488	7.24 ± 2.07	1	12	1371	6.96 ± 2.38	−2	12	0.037
Center	217	7.17 ± 2.09	2	12	906	6.97 ± 2.41	−1	12	-
South	296	7.05 ± 1.99	2	11	1263	6.56 ± 2.43	−3	12	0.002
Parents Education									
Non-university	692	6.97 ± 2.06	1	12	2155	6.52 ± 2.42	−3	12	0.000046
University	309	7.61 ± 1.95	3	12	1191	7.38 ± 2.31	−1	12	-

**Table 3:** Comparison of KIDMED Index answers between the periods 1998–2000 and 2019–2020.

KIDMED Test Items	Answers Scoring		1998–2000 *n* (%)	2019–2020 *n* (%)	χ2 *p*-Value
Takes a fruit or fruit juice every day	No	0	125 (12.5)	969 (27.4)	<0.001
	Yes	1	876 (87.5)	2571 (72.6)	
Has a second fruit every day	No	0	432 (43.2)	1730 (48.9)	0.001
	Yes	1	569 (56.8)	1810 (51.1)	
Has fresh or cooked vegetables regularly once a day	No	0	311 (31.1)	1253 (35.4)	-
	Yes	1	690 (68.9)	2287 (64.6)	
Has fresh or cooked vegetables more than once a day	No	0	746 (74.5)	2382 (67.3)	<0.001
	Yes	1	255 (25.5)	1158 (32.7)	
Consumes fish regularly (at least 2–3 times/week)	No	0	175 (17.5)	1339 (37.8)	<0.001
	Yes	1	826 (82.5)	2201 (62.2)	
Goes > 1 time/week to a fast-food restaurant	No	0	971 (97)	2747 (77.6)	<0.001
	Yes	−1	30 (3)	793 (22.4)	
Likes pulses and eats them >1 time/week	No	0	106 (10.6)	1060 (29.9)	<0.001
	Yes	1	895 (89.4)	2480 (70.1)	
Consumes pasta or rice almost every day (5 or more times per week)	No	0	668 (66.7)	1817 (51.3)	<0.001
	Yes	1	333 (33.3)	1723 (48.7)	
Has cereals or grains (bread, etc.) for breakfast	No	0	384 (38.4)	1268 (35.8)	-
	Yes	1	617 (61.6)	2272 (64.2)	
Consumes nuts regularly (at least 2–3 times/week)	No	0	578 (57.7)	1701 (48.1)	<0.001
	Yes	1	423 (42.3)	1839 (51.9)	
Uses olive oil at home	No	0	257 (25.7)	312 (8.8)	<0.001
	Yes	1	744 (74.3)	3228 (91.2)	
Has breakfast	No	−1	40 (4)	429 (12.1)	<0.001
	Yes	0	961 (96)	3111 (87.9)	
Has a dairy product for breakfast (yoghurt, milk, etc.)	No	0	96 (9.6)	628 (17.7)	<0.001
	Yes	1	905 (90.4)	2912 (82.3)	
Has commercially baked goods or pastries for breakfast	No	0	877 (87.6)	2414 (68.2)	<0.001
	Yes	−1	124 (12.4)	1126 (31.8)	
Takes two yoghurts and/or some cheese (40 g) daily	No	0	532 (53.1)	781 (22.1)	<0.001
	Yes	1	469 (46.9)	2759 (77.9)	
Takes sweets and candy several times every day	No	0	662 (66.1)	2788 (78.8)	<0.001
	Yes	−1	339 (33.9)	752 (21.2)	

**Table 4:** Comparison of single item KIDMED answers according to educational level and sex over the two study periods.

	Primary Education											Secondary Education									
	Male						Female					Male					Female				
	1998–2000			2019–2020		χ2	1998–2000		2019–2020		χ^2^	1998–2000		2019–2020		χ^2^	1998–2000		2019–2020		χ^2^
KIDMED Test Items		*n*	(%)	*n*	(%)	*p*	*n*	(%)	*n*	(%)	*p*	*n*	(%)	*n*	(%)	*p*	*n*	(%)	*n*	(%)	*p*
Takes a fruit or fruit juice every day	No	17	(8.7)	221	(24.6)		19	(10.1)	210	(22.9)		45	(14.6)	257	(31.7)		44	(14.3)	281	(30.7)	
	Yes	179	(91.3)	676	(75.4)	a	169	(89.9)	709	(77.1)	a	264	(85.4)	553	(68.3)	a	264	(85.7)	633	(69.3)	a
Has a second fruit every day	No	75	(38.3)	381	(42.5)		75	(39.9)	357	(38.8)		146	(47.2)	472	(58.3)		136	(44.2)	520	(56.9)	
	Yes	121	(61.7)	516	(57.5)		113	(60.1)	562	(61.2)		163	(52.8)	338	(41.7)	b	172	(55.8)	394	(43.1)	a
Has fresh or cooked vegetables regularly once a day	No	63	(32.1)	318	(35.5)		56	(29.8)	311	(33.8)		108	(35)	315	(38.9)		84	(27.3)	309	(33.8)	
	Yes	133	(67.9)	579	(64.5)		132	(70.2)	608	(66.2)		201	(65)	495	(61.1)		224	(72.7)	605	(66.2)	
Has fresh or cooked vegetables >1 time/day	No	130	(66.3)	572	(63.8)		147	(78.2)	571	(62.1)		206	(66.7)	589	(72.7)		263	(85.4)	650	(71.1)	
	Yes	66	(33.7)	325	(36.2)		41	(21.8)	348	(37.9)	a	103	(33.3)	221	(27.3)		45	(14.6)	264	(28.9)	a
Consumes fish regularly (at least 2–3 times/week)	No	42	(21.4)	310	(34.6)		28	(14.9)	312	(33.9)		54	(17.5)	308	(38)		51	(16.6)	409	(44.7)	
	Yes	154	(78.6)	587	(65.4)	a	160	(85.1)	607	(66.1)	a	255	(82.5)	502	(62)	a	257	(83.4)	505	(55.3)	a
Goes >1 time/week to a fast- food restaurant	No	196	(100)	714	(79.6)		185	(98.4)	722	(78.6)		294	(95.1)	607	(74.9)		296	(96.1)	704	(77)	
	Yes	1	(0)	183	(20.4)	a	3	(1.6)	197	(21.4)	a	15	(4.9)	203	(25.1)	a	12	(3.9)	210	(23)	a
Likes pulses and eats them >1 time/week	No	17	(8.7)	281	(31.3)		17	(9)	279	(30.4)		35	(11.3)	209	(25.8)		37	(12)	291	(31.8)	
	Yes	179	(91.3)	616	(68.7)	a	171	(91)	640	(69.6)	a	274	(88.7)	601	(74.2)	a	271	(88)	623	(68.2)	a
Consumes pasta or rice almost every day (≥5 times per week)	No	131	(66.8)	468	(52.2)		116	(61.7)	505	(55)		203	(65.7)	377	(46.5)		218	(70.8)	467	(51.1)	
	Yes	65	(33.2)	429	(47.8)	a	72	(38.3)	414	(45)		106	(34.3)	433	(53.5)	a	90	(29.2)	447	(48.9)	a
Has cereals or grains (bread, etc.) for breakfast	No	50	(25.5)	296	(33)		71	(37.8)	291	(31.7)		115	(37.2)	306	(37.8)		148	(48.1)	375	(41)	
	Yes	146	(74.5)	601	(67)		117	(62.2)	628	(68.3)		194	(62.8)	504	(62.2)		160	(51.9)	539	(59)	
Consumes nuts regularly (at least 2–3 times/week)	No	126	(64.3)	413	(46)		107	(56.9)	419	(45.6)		168	(54.4)	398	(49.1)		177	(57.5)	471	(51.5)	
	Yes	70	(35.7)	484	(54)	a	81	(43.1)	500	(54.4)		141	(45.6)	412	(50.9)		131	(42.5)	443	(48.5)	
Uses olive oil at home	No	44	(22.4)	128	(14.3)		54	(28.7)	100	(10.9)		86	(27.8)	55	(6.8)		73	(23.7)	29	(3.2)	
	Yes	152	(77.6)	769	(85.7)		134	(71.3)	819	(89.1)	a	223	(72.2)	755	(93.2)	a	235	(76.3)	885	(96.8)	a
Has breakfast	No	3	(1.5)	55	(6.1)		3	(1.6)	78	(8.5)		15	(4.9)	106	(13.1)		19	(6.2)	190	(20.8)	
	Yes	193	(98.5)	842	(93.9)		185	(98.4)	841	(91.5)	b	294	(95.1)	704	(86.9)	a	289	(93.8)	724	(79.2)	a
Has a dairy product for breakfast (yoghurt, milk, etc.)	No	10	(5.1)	134	(14.9)		14	(7.4)	144	(15.7)		30	(9.7)	134	(16.5)		42	(13.6)	216	(23.6)	
	Yes	186	(94.9)	763	(85.1)	a	174	(92.6)	775	(84.3)	c	279	(90.3)	676	(83.5)		266	(86.4)	698	(76.4)	a
Has commercially baked goods/pastries for breakfast	No	179	(91.3)	616	(68.7)		170	(90.4)	627	(68.2)		262	(84.8)	544	(67.2)		266	(86.4)	627	(68.6)	
	Yes	17	(8.7)	281	(31.3)	a	18	(9.6)	292	(31.8)	a	47	(15.2)	266	(32.8)	a	42	(13.6)	287	(31.4)	a
Takes two yoghurts and/or some cheese (40 g) daily	No	97	(49.5)	232	(25.9)		105	(55.9)	212	(23.1)		159	(51.5)	152	(18.8)		171	(55.5)	185	(20.2)	
	Yes	99	(50.5)	665	(74.1)	a	83	(44.1)	707	(76.9)	a	150	(48.5)	658	(81.2)	a	137	(44.5)	729	(79.8)	a
Takes sweets and candy several times every day	No	147	(75)	733	(81.7)		137	(72.9)	741	(80.6)		175	(56.6)	637	(78.6)		203	(65.9)	677	(74.1)	
	Yes	49	(25)	164	(18.3)		51	(27.1)	178	(19.4)		134	(43.4)	173	(21.4)	a	105	(34.1)	237	(25.9)	

a: *p* < 0.001 b: *p* = 0.001 c: *p* = 0.003.

## Data Availability

There are restrictions on the availability of data for this trial due to the signed consent agreements and around data sharing, which only allow access to external researchers for studies following the project purposes. Requestors wishing to access the trial data used in this study can make a request to sgomez@gasolfoundation.org.
